# Optical material properties affect detection of deformation of non-rigid rotating objects, but only slightly

**DOI:** 10.1167/jov.25.6.6

**Published:** 2025-05-12

**Authors:** Mitchell J. P. van Zuijlen, Yung-Hao Yang, Jan Jaap R. van Assen, Shin'ya Nishida

**Affiliations:** 1 Cognitive Informatics Lab, Graduate School of Informatics, Kyoto University, Kyoto, Japan; 2 Perceptual Intelligence Lab, Faculty of Industrial Design Engineering, Delft University of Technology, Delft, The Netherlands

**Keywords:** deformation, rigidity, material perception, transparent

## Abstract

Although rigid three-dimensional (3D) motion perception has been extensively studied, the visual detection of non-rigid 3D motion remains underexplored, particularly with regard to its interactions with material perception. In natural environments with various materials, image movements produced by geometry-dependent optical effects, such as diffuse shadings, specular highlights, and transparent glitters, impose computational challenges for accurately perceiving object deformation. This study examines how optical material properties influence human perception of non-rigid deformations. In a two-interval forced choice task, observers were shown a pair of rigid and non-rigid objects and asked to select the one that appeared more deformed. The object deformation varied across six intensity levels, and the stimuli included four materials (dotted matte, glossy, mirror, and transparent). We found that the material has only a small effect on deformation detection, with the threshold being slightly higher for transparent than other materials. The results remained the same regardless of the viewing angles, light field conditions (Experiment 1), and the deformation type (Experiment 2). These results show the robust capacity of the human visual system to perceive non-rigid object motion in complex natural visual environments.

## Introduction

The deformation of an object provides valuable information for humans. For example, from how a non- rigid object deforms, we can visually judge the mechanical properties of materials, such as softness and viscosity, and biological properties of living creatures, such as liveness and animacy (Assen, Barla, & Fleming, 2018, Assen, Nishida, & Fleming, 2020; Chang & Troje, 2008; Kawabe, 2017; Kawabe, Maruya, Fleming, & Nishida, 2015; Kılı¸c & Dövencioǧlu, 2024; Paulun, Schmidt, Assen, & Fleming, 2017; Schmidt, Hegele, & Fleming, 2017; Schmidt, Paulun, Assen, & Fleming , 2017). However, determining whether an object is rigid or deforming (non-rigid) can become more computationally challenging when it is in motion within a scene. Although extensive studies have been made on how the visual system estimates the 3D structure from image motion of rigid objects (Braunstein, Hoffman, & Pollick, 1990; Koenderink, 1986; Norman & Todd, 1993; Ullman, 1979), only a small number of studies have been made on the perception of non-rigid 3D motion (Choi, Feldman, & Singh, 2024; Hogervorst, Kappers, & Koenderink, 1996; Jain & Zaidi, 2011; Koerfer & Lappe, 2022; Perotti, Todd, & Norman, 1996; Todd, 1982). Furthermore, most of these exceptional studies used artificial stimuli made of dots. In natural scenes where a variety of optical processes produce the appearance of a variety of materials, the relationship between the 3D structure and image motion can be highly complex (Ullman, 1977). Specifically, the projective correspondence between the 3D structure and the two-dimensional (2D) image may hold for a cloud of dots and surface texture produced by modulation in albedo and color (Todd, 1985), but not for the image features produced by diffuse shading, smooth occluding contours (Norman & Todd, 1994), specular highlights of glossy materials, and the brilliant glitters of transparent materials (Todd & Norman, 2019). Even when a 3D object makes the same movement relative to the viewer, the image features move in their own unique way, so that image motion provides useful cues used for material perception (Doerschner, Kersten, & Schrater, 2011; Doerschner, Fleming, et al., 2011; Tamura, Higashi, & Nakauchi, 2018; Yilmaz & Doerschner, 2014). Therefore reliably detecting deformation despite large changes in material properties is computationally challenging. Past studies have attempted to reveal how material property affects shape perception (Dövencioǧlu, Wijntjes, Ben-Shahar, & Doerschner, 2015; Dövencioǧlu, Ben-Shahar, Barla, & Doerschner, 2017; Fleming, Torralba, & Adelson, 2004; Fleming, Holtmann-Rice, & Bulthoff, 2011; Khang, Koenderink, & Kappers, 2007), but they only considered rigid objects.

In this article, we try to answer how well human observers can visually detect the deformation of a moving object, and how much their performance is affected by the optical material properties of the object. Investigation of this ability, which should involve the three-way interactions among material, shape, and motion, would provide insights into the fundamental abilities of human visual computation to estimate stable 3D structures while taking into account complex optics in real-world image formation. One possible strategy for human vision to detect an object's deformation is to test whether the stimulus contains deviations from the image changes expected to be produced by a rigid movement of the object. The image change of rigid motion differs for different optical features. The pattern of image changes is more complex, incoherent, and rapid for specular highlights or transparent glitters than for matte surface textures, shading, and occluding contours (Doerschner, Kersten, & Schrater, 2011; Tamura et al., 2018; Yildiran, Storrs, Fleming, & Doerschner-Boyaci, 2023). We therefore expected that deformation detection might be more difficult for glossy and transparent objects than for more typical matte and textured objects.

Note also that, to the best of our knowledge, few studies have been conducted on the deformation detection of realistic natural objects in computer vision. Realism leads to complex optical interactions with the object's shape and its surroundings. Accurately estimating such motions requires a deeper understanding of the scene, including heuristic approximations of physical laws and estimation of intrinsic material properties. Inconsistent pixel intensities, abrupt changes in motion fields, and layered motion cues make identifying correspondences between frames particularly challenging.

In a series of psychophysical experiments, we investigate the human visual system's sensitivity to detect deformation for various optical conditions, and to what extent the optical parameters of material affect the sensitivity. In partial agreement with our expectation, our results show that the deformation detection sensitivity was lower for a transparent object than for the other objects. The difference, however, was small. The deformation detection sensitivity for glossy materials was similar to that for matte materials. In general, our results show that deformation detection is stable despite significant changes in image motion due to material changes, suggesting the exceptional ability of the human visual system to recognize 3D structures of dynamic natural scenes stably.

## Experiment 1

Experiment 1 examined how the deformation detection sensitivity is affected by material while changing the viewing angle and light field.

### Methods

The experiments were performed in accordance with the Declaration of Helsinki and approved by the Research Ethics Committee of the Graduate School of Informatics, Kyoto University (approval no. KUIS-EAR-2020-003). The experiment has been pre-registered with the Open Science Framework (OSF, https://osf.io/u7qs5).

#### Participants

Twenty-five Kyoto University students above the age of 20 participated in the experiment. They were recruited through the Kyoto University recruitment system and were compensated for their participation. All participants had normal or corrected-to-normal vision and provided informed consent before participating in the experiments.

#### Apparatus and stimuli

The experiment was performed on a calibrated Eizo ColorEdge CG303W display (refresh rate: 60Hz, resolution: 1920 × 1200 pixels, viewing distance: 65 cm) using Psychtoolbox-3 (Brainard, 1997 ) with Matlab R2021b. All stimuli (512 × 512 pixels) were presented at an approximately 15° visual angle.

To study the human perception of deformation, we utilized computer-rendered stimuli to create a dataset of rigid and deforming objects. These stimuli consisted of a rotating infinite-knot object with different degrees of deformation. This shape is interesting because it self-occludes and casts shadows on itself. The base shape for all stimuli is a high-resolution infinite-knot object mesh (simply referred to as “knot” hereafter), which is one of the default geometries in Maxwell 4 (Next Limit Technologies). This particular knot comprises 184,000 triangles, ensuring that the level of detail is exceptionally high. As a result, even when deformations are applied to the shape, the surface of the stimuli retains its smoothness. The stimuli were also rendered at different viewing angles under various light field conditions with diverse optical material appearances. We introduced the details of these manipulations in the following paragraph.

##### Squeeze deformations

**Figure 1.**
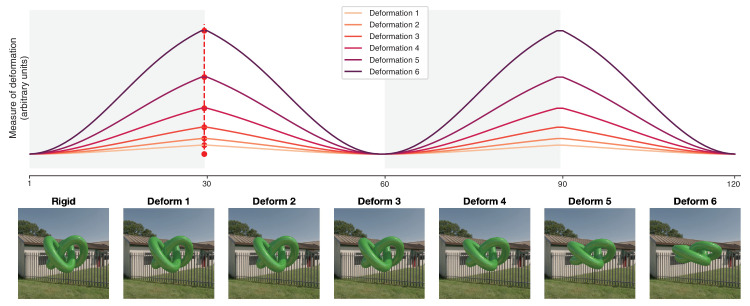
The deformation intensities. The top panel shows the rate of deformation for each of the deformation intensities as a function of the frames. The deformation images for the first 30 frames (left of the red-dotted line) were simulated using RealFlow 10.5 (Next Limit Technologies). The remaining 90 frames were copies of these original 30 frames with regard to the object shape. That is, the deformation intensity in frame 31 was identical to 30, frame 32 was identical to frame 29, etc. The bottom panel shows the maximum deformations in each deformation intensity at the 30th frame (red dotted). We rendered a 120-frame movie in which two repetitions of inward-outward deformations were combined with the rotation of the object around its vertical axis, either clockwise or counterclockwise, at the speed of 360°/120 frames. Each stimulus presentation in the main experiments consisted of a 30-frame (500-ms) movie cut from the 120-frame movie, starting at either frame 1, 31, 61, or 91.

To simulate deformations on the knot object, we used RealFlow 10.5 (Next Limit Technologies). We applied a horizontal inward pulling force, which would “squeeze” the knot object inwards along its vertical axis. We used 6 logarithmically increasing force values (3, 5.27, 9.24, 16.23, 28.48, 50) to generate six deformation intensity conditions, corresponding to six sets of 30 frames. The force remained constant within each condition. The six logarithmic force intensities resulted in six logarithmically spaced deformations in the object's geometry, where the magnitude of deformation was measured as the square root of the summed squared geometric distances between paired vertices. For each intensity, we rendered 30 frames in which the knot object deformed from a non-deformed shape at the first frame, to the point of maximum deformation at frame 30. Thus the first frame would be identical in shape deformation across all six force intensities. We duplicated the 30 frames to create a total of 120 frames for each force intensity, in which the deformation would gradually increase for the first 30 frames, then reverse the deformation direction for the next 30 frames. In addition to the six deformation intensities, we created a non-deforming, rigid condition, where we simply showed the default knot object for 120 frames. See Figure 1 for a visual representation of this (Top panel) and a static representation of the maximum deformations per force intensity (Bottom panel). A dynamic example of the stimuli can be found in the supplementary materials (Supplemental Video S1).

Next, we rendered the seven sets of 120 frames discussed above in Maxwell Rendered 4 (Next Limit Technologies), and each of the frames was a 512 × 512 pixels image. We rotated the objects within each set with a 3° yaw angle per frame (i.e., rotation around the vertical axis), resulting in one complete rotation of 360° per 120 frames. Without the rotational motion, it would be trivial to detect deformation in the object, as any perceived motion would indicate deformation and our intended deformation perception experiment would simply become generic motion perception instead.

##### Viewing angles

**Figure 2.**
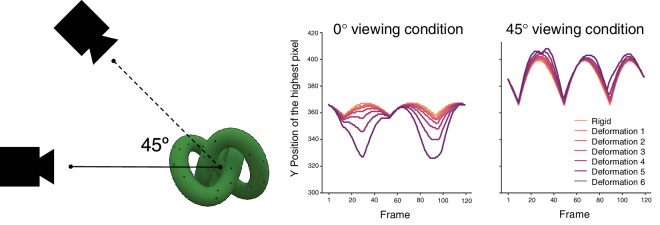
The two viewing angles. The left panel showed how the virtual camera viewed the object in the image rendering. The distance from the viewpoint remained consistent between these two viewing conditions. The middle and right panels showed how the highest pixel position changes across frames in different deformation under 0° viewing conditions and 45° viewing conditions.

We used two viewing angle conditions: 0° and 45° (Figure 2). In the 0° viewing condition, the camera viewpoint was set at the same height as the knot object that rotated around the vertical axis. Thus, the stimuli were presented from a viewpoint exactly in the middle of the stimuli, and the deformation was applied in such a way that the top and bottom of the stimuli were compressed symmetrically toward the center. As a result, to detect deformation, the observers could take a simple strategy of tracking the top and bottom heights of the stimuli. To illustrate this, in Figure 2 (middle panel), we show the position of the highest visible pixel (please note that the lowest pixel, effectively mirrored, is not visualized). This single pixel had the potential to predict deformation intensity perfectly, regardless of optical parameters. In response to the possible concern about this potentially material- and illumination-invariant trivial image cue, we included the 45° viewing condition, in which, all stimuli were rendered from a viewpoint 45° below the original 0° viewing perspective whereas the look-at point at the center of the object stayed constant. Importantly, the distance from the viewpoint or camera remained consistent between these two viewing conditions, as depicted in Figure 2 (left panel). As depicted in Figure 2 (right panel), the informativeness of this single pixel was diminished across different deformation intensities in the 45° viewing condition. This adjustment was made to heighten the level of task difficulty and reduce reliance on specific response strategies compared to the 0° viewing condition.

##### Light fields

**Figure 3.**
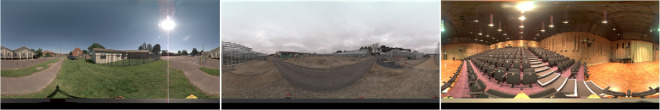
The three light fields in the Experiment 1. From left to right, we have labeled these as Sunny, Overcast, and Indoor.

We selected three light fields from the SYNS dataset (Adams et al., 2016), which contains high dynamic range (HDR) panoramic images from a diverse set of approximately 100 locations. We computed the distances within the spherical harmonics space between each pair of HDR images. Next, we picked the triplet of HDR images that were most distant. By doing so, we attempted to select the three illuminations that were the most distinct, with the intention to capture the largest visual variation in our set of HDR images. We then used these three HDR images as illumination maps during rendering by using the Image Based Lighting technique from Maxwell, which is intended to make it appear as if the rendered object is within the environment captured by the HDR image. The three selected HDR images were no. 7, no. 73, and no. 89 from the SYNS dataset, which we have relabeled as sunny, overcast, and indoor, respectively, which are visualized in a standard dynamic range in Figure 3.

##### Materials

**Figure 4.**
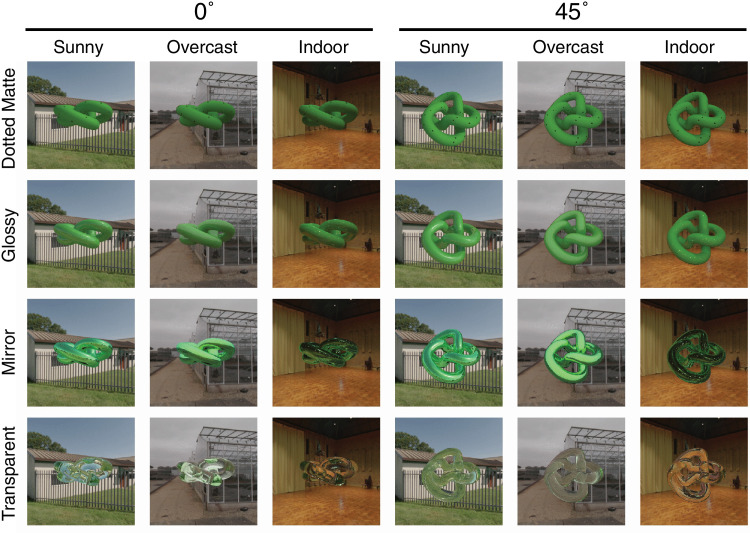
The stimuli in the Experiment 1. Two viewing angles (0° and 45°) are displayed on the left 12 panels and the right 12 panels, respectively. Four materials (Dotted Matte, Glossy, Mirror, and Transparent) are displayed in panels going from top to bottom. Three illumination light fields (Sunny, Overcast, and Indoor) can be seen in the panels from left to right.

Last, we rendered the objects with a variety of optical materials. We used the term “optical material” to indicate that only the optical parameters of the stimuli change as a response to material parameter changes. Outside the digital realm, a material change would virtually always lead to geometry changes (i.e., the microstructure of the [sub-]surface). These changes to the microstructure are, in essence, the cause of the changes in appearance. With digital renderings, we can cause the appearance change without altering the geometry. The material parameters were set manually within Maxwell, drawing on Maxwell presets when available. One consistency across materials is that we set the base color of all materials to green. Furthermore, using the same deforming knot across materials ensured consistency and provided a common baseline, allowing us to isolate the effects of material properties on structure-from-motion. We created a set of four materials: dotted matte, glossy, mirror and transparent (reflective index: water). The dotted matte material was selected because we speculate the dot texture might function as a material and illumination invariant cue. Although the dot density may appear low, it remained continuously visible during rotation. The glossy material was chosen for its ability to create optical highlights that emphasize shape, movement, and light interactions. The mirror material was included as the surface appearance is nearly completely dependent on the illumination, similar to the transparent material, which was included as its appearance dependencies also include a refractive component. Figure 4 shows the four materials rendered in each of the three illumination scenes (i.e., sunny, overcast, and indoor). Dynamic examples of the stimuli are available in the supplementary materials (Supplementary Video S2). In addition to the four materials we selected, we also tested six additional materials in a preliminary experiment. The results showed only small differences in deformation detection performances between the selected four and the remaining six. See Supplementary Appendix A for more details.

#### Design

The independent variables included two viewing angles (0° and 45°), three light fields (sunny, overcast, and indoor), four materials (dotted matte, glossy, mirror, and transparent), and six deformation intensities (1-6 range of deformations). The above 144 conditions were repeated eight times for counterbalance with three nuisance variables, including (1) rotation direction of the stimuli (clockwise or counterclockwise), (2) order of target presenting intervals (first or second interval), and (3) the starting frame of each stimulus (Frame 1/61 or Frame 31/91 for inward and outward deformations, respectively, see Figure 1). For each stimulus presentation, 30 frames were selected from 120 frames. The starting frame was either the minimum deformation frame (frame 1 or 61) or the maximum deformation frame (frame 31, 91), and randomly selected from the four. The order of the frames (normal or reversed) was randomly determined. In the normal order, the stimuli were observed rotating clockwise, although they rotated counterclockwise in the reversed order. As such, each of the three nuisance variables has two levels, which we counterbalanced with eight repetitions per unique condition, 1152 main trials in total. Additionally, 192 catch trials with rigid, non-deforming stimuli were included (four materials × two viewing angles × three illuminations × eight repetitions). This rigid condition was mainly for checking the response bias of the participants. Because of system memory limitations (which were addressed before conducting the main experiments), it was not possible to load all the images before the start of the experiment. To address this, we split the 1344 trials in total into six blocks of 224 trials each and loaded the required images for each block into memory during a 45-second break between blocks.

### Procedure

Across all experiments, participants were seated in a basement of Kyoto University. They were given instructions that the experiment aimed to investigate how people perceive changes in the shape of materials or objects, which we referred to as “deformation.” It was explicitly mentioned that the term “deformation” did not encompass rotations. To clarify this, we presented participants with an example of the stimuli featuring the most significant deformation in a short training session. Additionally, we emphasized that the deformations in the task could be considerably more subtle than the example provided. We used a two-interval forced choice (2-IFC) task, where participants were shown a reference and a target stimulus in each trial. Both stimuli were presented at 60 frames per second, for a stimulus presentation time of 0.5 seconds, and were separated by a 0.5-second interstimuli interval (ISI). The target stimuli would be presented at one of the seven deformation intensities (including 0 for catch trial to check the response biases), and the reference stimuli would always be presented at deformation intensity 0. The other stimulus conditions for the reference stimuli were always identical to those for the target stimuli (i.e., light field, material, viewing angle, rotation direction). As such, the target and reference could only vary on deformation intensity. The task was to select the stimulus that was deforming and the feedback was not given to the participants.

In a preliminary experiment (Appendix A), in addition to the 2-IAFC task, we tested a “Yes-No task”, in which the observers judged whether the target stimulus (presented alone in a trial without being accompanied by the reference stimulus) appeared deformed or not. The results were consistent with those obtained with the 2-IAFC task. In the main experiments, we used the latter method only, because it would be more robust against criterion shifts.

### Results

**Figure 5.**
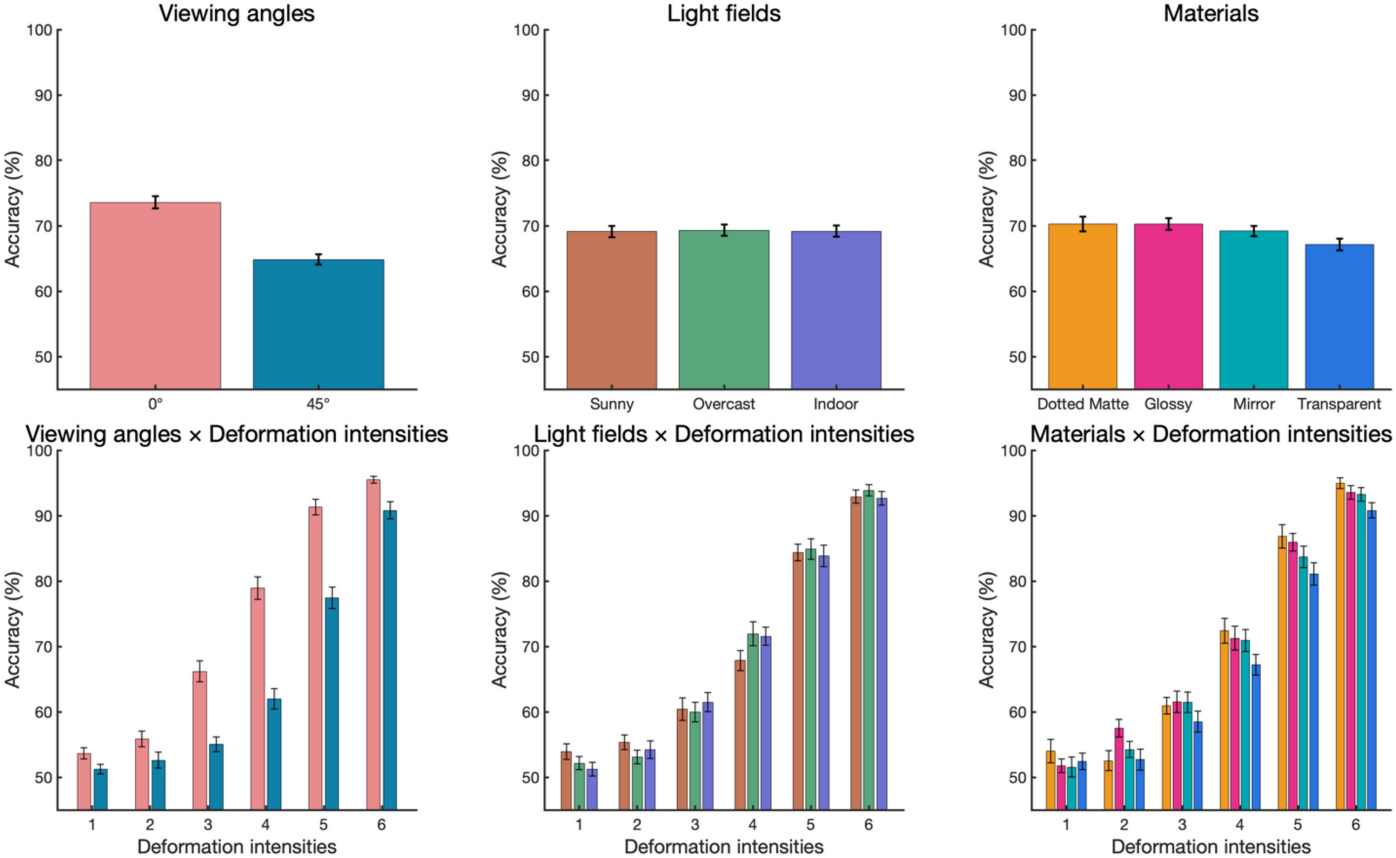
The accuracy in Experiment 1. (**a**) From left to right, panels show the main effects of the viewing angles, light fields, and materials. (**b**) Those variables as functions of deformation intensities.

Figure 5 shows how the accuracy of discriminating rigid versus deformation was affected by the viewing angles, light fields, and materials, either as a whole or separately for each deformation intensity. Note that “accuracy” here refers to the proportion of trials in which participants perceived greater deformation, influenced by perceptual biases introduced by specular flow in computer-generated stimuli. We analyzed the results in two ways. One was to use psychometric function fitting to estimate the threshold deformation magnitude, whereas the other was to use the generalized linear mixed model (GLMM) using accuracy as the dependent variable to test main effects and interactions among factors.

#### Psychometric function fitting for thresholds comparison

**Figure 6.**
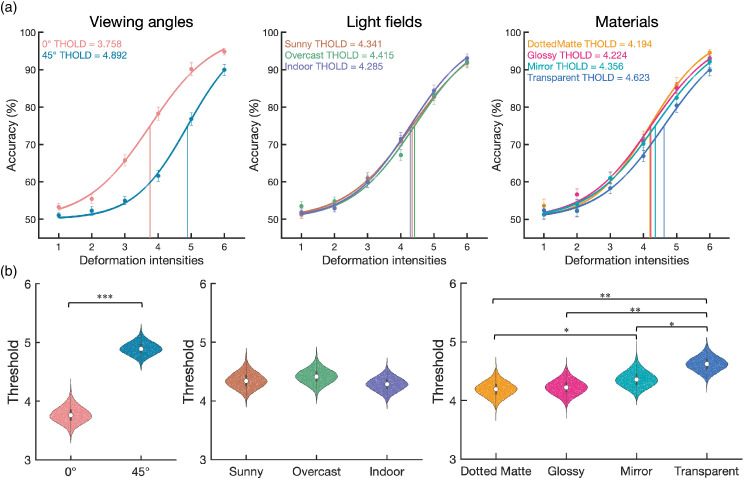
The fitting curves and the bootstrap-generated distribution of the threshold in Experiment 1 (The main effect). (**a**) The fitting curves based on the median of the threshold and slope of 10,000 repetitions by the bootstrapping method. From left to right, panels show the viewing angles, light fields, and materials, respectively. The median threshold is shown in the top-left corner. (**b**) The distributions of the threshold in each condition estimated by the bootstrapping method. The black bar and white dot inside the violin plot indicate the interquartile range [Q1,Q3] and median [Q2], respectively. For threshold difference, *** *p* < 0.001; ** *p* < 0.01; * *p* < 0.05 after Bonferroni-Holm correction.

Psychometric curves were estimated by fitting logistic functions using the quickpsy R package. The model included two parameters: a variable slope and an intercept. The slope is the logistic growth rate of the function to predict performance as a function of deformation intensity, and the intercept represents the deformation intensity at which the model predicts 50% performance. The threshold for each condition was defined by the deformation intensity giving 75% accuracy. To statistically compare the deformation thresholds among conditions, we used the bootstrapping method to create simulated sampling distributions of the threshold (10,000 samples). In each repetition, we resampled the original participants with replacements and used group-level curve fitting to get the slope and threshold in each condition. The fitting curves (based on the median of the threshold and slope of 10,000 repetitions) and the distribution of the threshold in each condition are shown in Figure 6. To evaluate the threshold difference between each pair of conditions (e.g., glossy vs. transparent), we calculated the distributions of the threshold difference using Bonferroni-Holm correction for multiple comparisons.

Regarding the effect of viewing angle, the 45° viewing (*Median* = 4.89) has a significantly higher in threshold than the 0° viewing (*Median* = 3.76, *p* < 0.0001) condition. Regarding the effect of material, transparent (*Median* = 4.62) was significantly higher in threshold than dotted matte (*Median* = 4.19, *p* = 0.0018), glossy (*Median* = 4.22, *p* = 0.0018), and mirror (*Median* = 4.36, *p* = 0.0148), and the mirror was higher than dotted matte (*p* = 0.0330). There was no significant difference among the light field conditions (*p*s > 0.1890).

**Figure 7.**
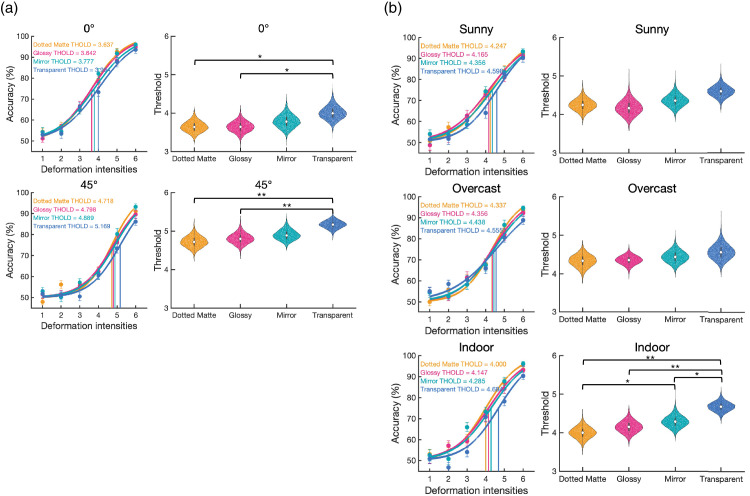
The fitting curves and the bootstrap-generated distribution of the threshold in Experiment 1 (The interaction effect). (**a**) the simple main effect of materials within each viewing angle. (**b**) the simple main effect of materials within each light field. The left columns in panels (**a**) and (**b**) show the fitting curves based on the median of the threshold and slope of 10,000 repetitions by the bootstrapping method. The median threshold is shown in the top-left corner. The right columns in panels (**a**) and (**b**) show the distributions of the threshold in each condition estimated by the bootstrapping method. The black bar and white dot inside the violin plot indicate the interquartile range [Q1,Q3] and median [Q2], respectively. For threshold difference, *** *p* < 0.001; ** *p* < 0.01; * *p* < 0.05 after Bonferroni-Holm correction.

Additionally, because we were interested in whether the viewing angle and light field affected the effects of the material, we also fitted the functions separately. Regarding the simple main effect of materials within each viewing angle (Figure 7a), the transparent is significantly higher in threshold than dotted matte (0° viewing: *p* = 0.0204; 45° viewing: *p* = 0.0100) and glossy (0° viewing: *p* = 0.0255; 45° viewing: *p* = 0.0054) in 0° viewing and 45° viewing conditions, respectively. Regarding the simple main effect of materials within each light field (Figure 7b), only the indoor condition showed that the transparent (*Median* = 4.68) has significantly higher in threshold than dotted matte (*p* = 0.0018), glossy (*p* = 0.0020) and mirror (*p* = 0.0456), and mirror is higher than dotted matte (*p* = 0.0456). There was no significant difference among materials in other light field conditions (*p*s > 0.0576).

#### Generalized linear mixed model for accuracy analysis

In order to examine higher-order interactions, we analyzed the same data by a GLMM using the lme4 and emmeans in R packages. A GLMM logistic regression considered the fixed effects of deformation intensity (continuous variable), viewing angle, light field and material, and all of their higher-order interactions. The model also included a random intercept and slope per participant.

As summarized in Table 1, the model found significant main effects of deformation intensity (*χ*^2^ = 360.44, *p* < 0.0001), viewing angle (*χ*^2^ = 109.92, *p* < 0.0001), and material (*χ*^2^ = 12.43, *p* = 0.0061), but not that of light field (*χ^2^* = 0.35, *p* = 0.8402). Regarding the effect of material, Tukey's HSD post hoc test suggested that the transparent material was significantly different from the dotted matte (*z* = 3.93, *p* = 0.0005), glossy (*z* = 3.95, *p* = 0.0005), and mirror (*z* = 2.62, *p* = 0.0435).

**Table 1.** Summary of the GLMM. The table contains the main effects, two-way interactions, as well as higher-order interactions. *** *p* < 0.001; ** *p* < 0.01; * *p* < 0.05.

Factor	!!	*df*	*P*
Experiment 1			
Deformation intensity	360.443	1	<0.0001***
Viewing angle	109.922	1	<0.0001***
Light field	0.348	2	0.8402
Material	12.427	3	0.0061**
Deformation intensity: viewing angle	106.805	1	<0.0001***
Deformation intensity: light field	5.383	2	0.0678
Deformation intensity: material	18.691	3	0.0003***
Viewing angle: light field	2.370	2	0.3057
Viewing angle: material	0.630	3	0.8894
Light field: material	11.824	6	0.0660
Deformation intensity: viewing angle: light field	0.847	2	0.6546
Deformation intensity: viewing angle: material	0.073	3	0.9948
Deformation intensity: light field: material	4.752	6	0.5760
Viewing angle: light field: material	7.869	6	0.2479
Deformation intensity: viewing angle: light field: material	2.587	6	0.8586
Experiment 2			
Deformation intensity	176.875	1	<0.0001***
Viewing angle	87.175	1	<0.0001***
Deformation type	62.505	2	<0.0001***
Material	14.432	3	0.0024**
Deformation intensity: viewing angle	92.170	1	<0.0001***
Deformation intensity: deformation type	80.808	2	<0.0001***
Deformation intensity: material	13.877	3	0.0031**
Viewing angle: deformation type	16.553	2	0.0003***
Viewing angle: material	2.661	3	0.4469
Material: deformation type	10.032	6	0.1233
Deformation intensity: viewing angle: deformation type	5.534	2	0.0629
Deformation intensity: viewing angle: material	1.412	3	0.7027
Deformation intensity: deformation type: material	2.550	6	0.8628
Viewing angle: deformation type: material	10.245	6	0.1147
Deformation intensity: viewing angle: deformation type: material	4.296	6	0.6367

There was a significant interaction between deformation intensity and viewing angle (*χ*^2^ = 106.81, *p* < 0.0001), and Tukey's HSD post hoc test between slopes estimated from the deformation intensity (because of the continuous variable) suggested that 45° viewing condition has a steeper slope than the 0° viewing condition (*z* = 10.37, *p* < 0.0001). There was also a significant interaction between deformation intensity and material (*χ*^2^ = 18.69, *p* = 0.0003). The Tukey's HSD post hoc test showed that the slope as a function of deformation intensity was significantly shallower for the transparent material than for the dotted matte (*z* = 4.15, *p* = 0.0002) and glossy materials (*z* = 3.02, *p* = 0.0137). There was no other two-way nor higher-level interaction (see Table 1 for a GLMM summary).

### Discussion

We found that the different light fields had little effect on distortion detection. It is important to note that the three light conditions (sunny, overcast, indoor) used in the experiment were selected as the most distinct images from the SYNS dataset. Even across these distinct illumination scenes, not finding an effect of light field implies that the human visual system is robust against illumination effects for the perception of deformation.

On the other hand, the viewing angle had a significant effect on the deformation detection threshold. This effect was expected for our stimuli, due to the relationship between the distortion force direction and the viewing angle. The image changes produced by deformation, including changes in the object height, are more evident in the 0° viewing condition than for the 45° viewing condition (Figure 2).

The primary focus of this study is the effect of material on deformation detection and how it interacts with other elements. The results showed that although material had a significant effect on deformation detection, the impact was relatively minor. The performance is best for the dotted matte material and similarly good for glossy. Mirror was slightly worse, and transparent performed worst, but the threshold difference between dotted matte and transparent was only 8.58% in terms of our deformation scale.

Our selection of materials was somewhat more arbitrary than the selection of light field, but we are confident that our selection of materials consists of visually distinct materials, including matte, glossy, reflective, and refractive materials. Only finding a minor effect of these distinct materials further implies that the human visual system is robust against optical effects for the perception of deformation. In addition, the effects of the material are unaffected by light field, or by viewing angle. Note that the effect of material on deformation detection was similarly minor even for the 45° viewing condition, where simple 2D image cues of deformation are much less evident than the 0° viewing condition.

## Experiment 2

Experiment 1 used only one type of deformation. In this experiment, we introduced two new types of deformation to test the robustness of our findings.

### Methods

#### Participants

Another group of 25 Kyoto University students above the age of 20 participated in the study. None of them participated in the previous experiment.

#### Stimuli

**Figure 8.**
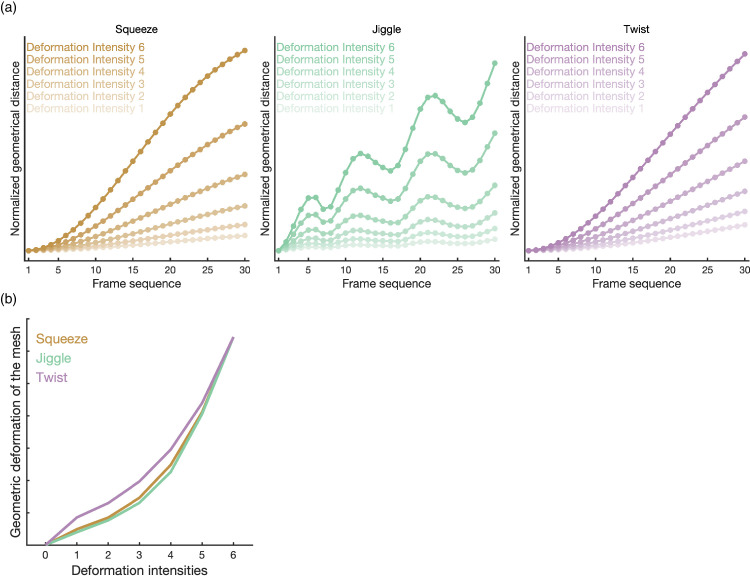
The three deformation types used in Experiment 2. (**a**) The time course of the distortion magnitude for the three deformation types. The distortion magnitude is the geometrical distance in the x-y-z coordinates of the corresponding points between a given frame and the first rigid frame. (**b**) The effect of the applied force on the shapes for the three deformation types.

As in the previous experiment, stimuli were rendered with the four materials (i.e., dotted matte, glossy, mirror, and transparent) in the two viewing angles (i.e., 0° viewing and 45° viewing). All stimuli were generated only under the sunny light field. In addition to the previous “squeeze” deformation type (i.e., the force being applied along the vertical axis from below and from above making it appear to be squeezed along this axis; see 2.1.2 squeeze deformations), we added two new types of deformation called “jiggle” and “twist.” All three deformation types were simulated by RealFlow 10.5 (Next Limit Technologies). We again created six levels of logarithmically increasing maximum forces that were used to simulate six levels of deformation intensity. For each maximum force intensity, we rendered 30 frames in which the knot object deformed, starting at a rigid, non-deformed shape. Across all deformation intensities and across all deformation types, the shape of the starting frame was identical. See Figure 8a for a visualization of the deformation intensity across the frames for the three types of deformations, normalized across deformation types. The stimuli were rendered and presented without a visible background (i.e., presented on a uniform dark background), and dynamic examples of the stimuli are available in the supplementary materials (Supplementary Video S3).

##### Jiggle deformation

For the previously used Squeeze deformation, the stimuli would display an increasing amount of deformation during these 30 frames. This was not the case for the new Jiggle deformation, where the direction of the force (pushing or pulling) would flip every five frames (i.e., a pulling force for five frames would change into a pushing force for five frames, only to change back again, etc.). The force was centered above the stimuli, with a spherical fall-off field,^1^ where the border of the spherical fall-off field was exactly at the center of the deforming object. To keep the whole object itself from simply translating up and down in a non-deforming method, we added a very strong drag force to keep the bottom half of the object from moving, while allowing the top half to deform. This rapidly alternating pulling/pushing force from above would generate a deformation similar to a jiggling object.

##### Twist deformation

For the twist deformation, we placed a rotating force “vortex” above the object, with a spherical fall-off field, similar to the jiggle deformation. The rotating force was of constant intensity across the duration of the simulation (but that constant intensity, of course, changed across deformation intensity conditions). We also placed a drag force near the bottom of the stimuli, to prevent the whole object from rigidly rotating.

##### Applied maximum forces across deformation intensities

In addition to the absolute intensity of the force applied to the object, the 3D arrangement of the force(s) and the object being deformed, are also important in determining the final outcome. We decided to include it for the sake of completion and to illustrate our workflow. We initially tried to keep the absolute forces applied across deformation types constant, but found no arrangement of object and forces in which the same forces would result in acceptable deforming stimuli, as the forces would for example barely deform the object, or deform it to absurd amounts. We next tried different absolute forces, but with a constant logarithmic ratio across deformation types. Specifically, we tried 1 to 10 in 6 logarithmic steps for Twist and 1 through 15 in 6 logarithmic steps. We found that small tweaks were required to better match the ratios of the absolute geometric deformation across the novel deformation types to the original deformation type. The final forces used were 1, 1.98, 3.4, 5.84, 10.04, and 15 for jiggle and 1, 1.58, 2.51, 3.98, 6.31, and 10 for twist. For the rigid stimuli, a force of 0 was, of course, used in both cases. See Figure 8b for a visualization of these forces, normalized to better show the similar ratios, along with the original Squeeze deformation.

#### Design and procedure

The procedure was identical to that of the previous experiment. The independent variables included two viewing angles (0° viewing and 45° viewing conditions), three deformation types (squeeze, jiggle, and twist), four materials (dotted matte, glossy, mirror, and transparent), and six deformation intensities (1-6 range of deformations). The above 144 conditions were repeated eight times for counterbalance with three nuisance variables (rotation direction × target presenting intervals × starting frame), resulting in 1152 trials in total. Additionally, 192 catch trials (two viewing angles × three deformation types × four materials × eight repetitions) with rigid, non-deforming stimuli were included. The second experiment was not pre-registered but used a similar experimental design and analysis methods as the first.

### Results

**Figure 9.**
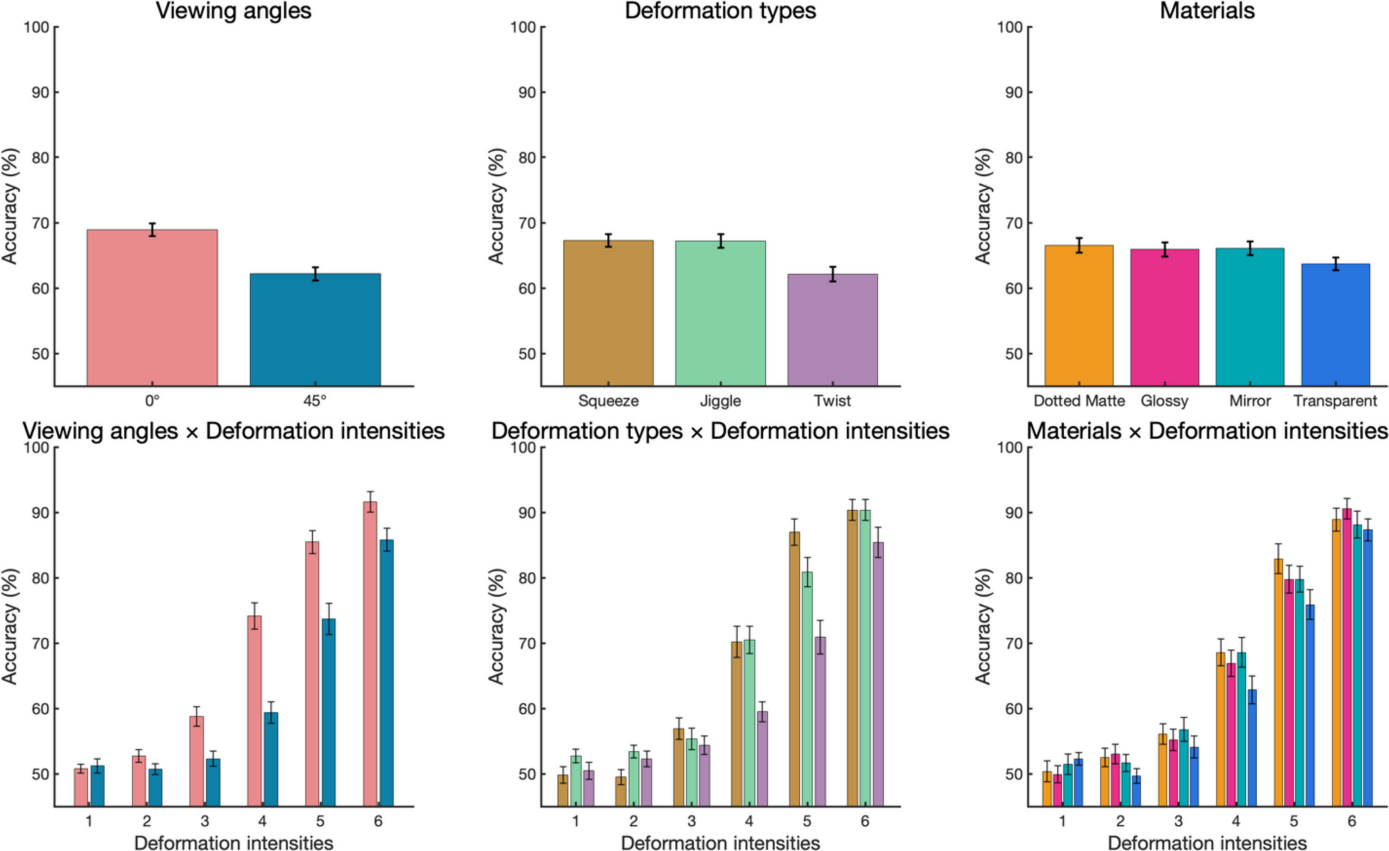
The accuracy in Experiment 2. (**a**) From left to right panels showed the main effects of viewing angles, deformation types, and materials. (**b**) Those variables as functions of deformation intensities.

One participant was removed from further analysis due to the exceptionally low performance (accuracy = 37.94%, cf., group-averaged accuracy = 65.55%). The result suggests that this participant might have misunderstood the instruction. The accuracy and the accuracy as the function of deformation intensities in each condition are shown in Figure 9. In general, we found similar results to those found in the previous experiment (i.e., the accuracy of deformation detection improves with an increase in the magnitude of the deformation).

#### Psychometric function fitting for threshold comparison

**Figure 10.**
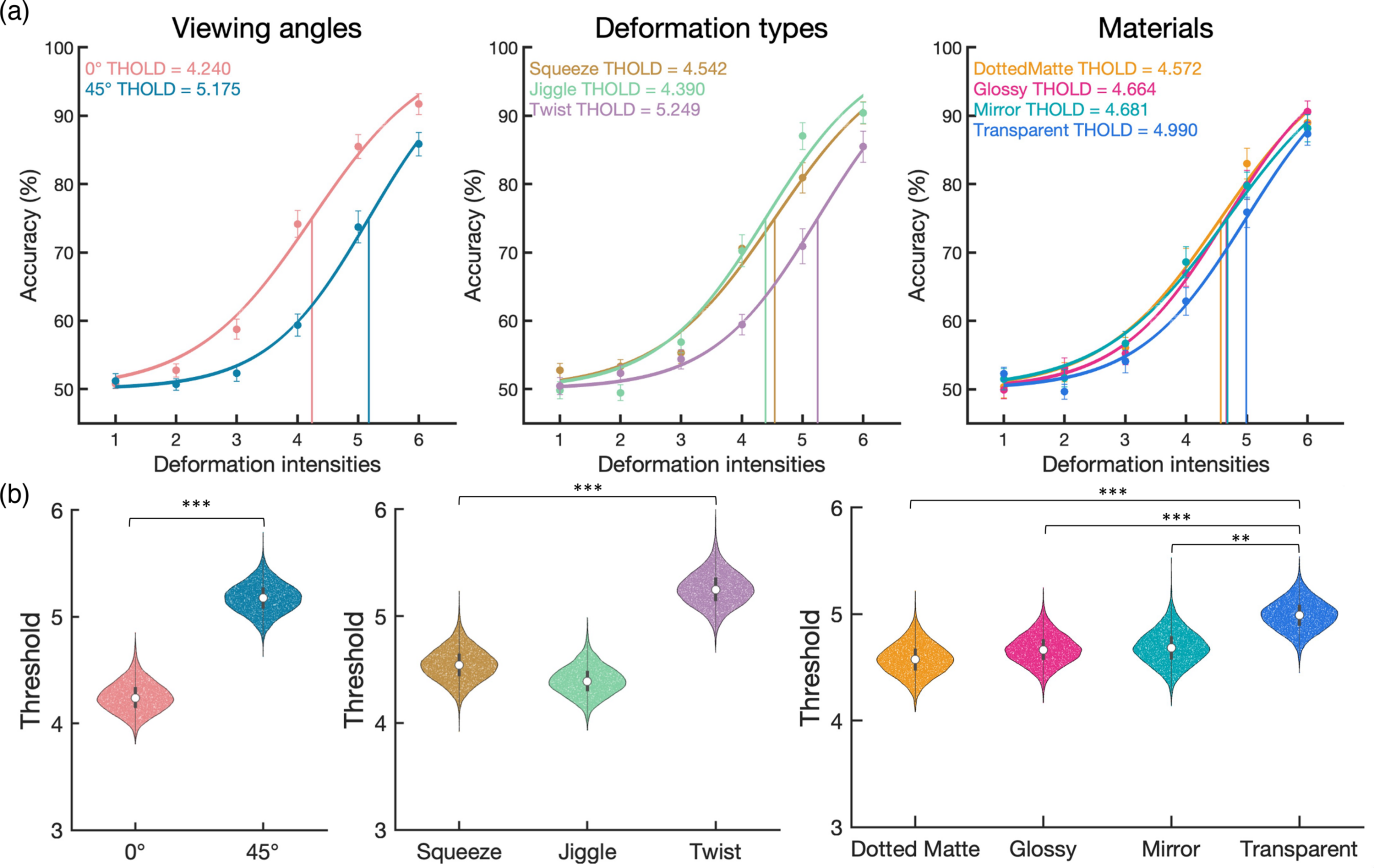
The fitting curves and the bootstrap-generated distributions of the threshold in Experiment 2 (The main effect). (**a**) The fitting curves were based on the median of the threshold and slope of 10,000 repetitions from the bootstrapping method. From left to right panels show the viewing angles, deformation types, and materials. The values in the top-left corner indicate the threshold median in each condition from Bootstrapping. (**b**) The simulated distributions of the threshold in each condition from the bootstrapping method. The center black bars and white dots inside the violin plots indicate the interquartile range [Q1,Q3] and median [Q2]. *** *p* < 0.001; ** *p* < 0.01; * *p* < 0.05 after Bonferroni-Holm correction. (**b**) the simple main effect of materials within each viewing angle. (**c**) the simple main effect of materials within each deformation type.

The fitting curves and bootstrap-generated distributions of the threshold are shown in Figure 10. The results were comparable with Experiment 1. The threshold comparison of viewing angle shows that the 45° viewing condition (*Median* = 5.18) has a significant higher threshold than 0° viewing condition (*Median* = 4.24, *p* < 0.0001). There were significant differences of threshold comparisons in material, where the transparent material (*Median* = 4.99) is higher in threshold than dotted matte (*Median* = 4.57, *p* < 0.0001), Glossy (*Median* = 4.66, *p* < 0.0001) and Mirror (*Median* = 4.68, *p* = 0.0048). The twist deformation type (*Median* = 5.25) showed higher threshold than the squeeze (*Median* = 4.54, *p* < 0.0001) and jiggle (*Median* = 4.39, *p* < 0.0001).

**Figure 11.**
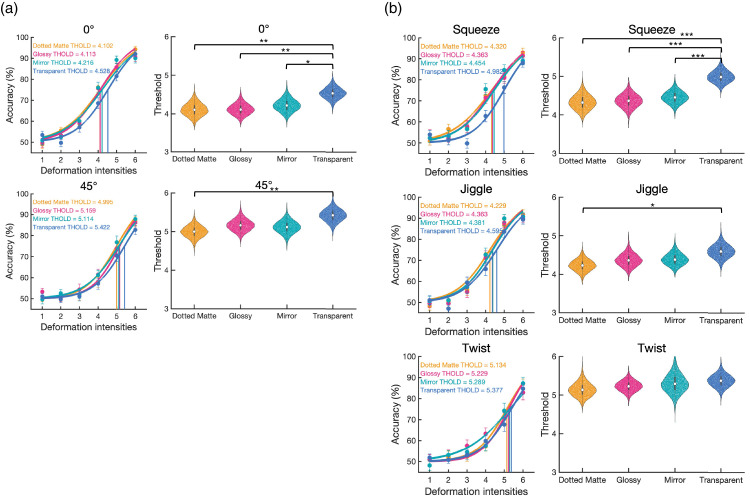
The fitting curves and the bootstrap-generated distribution of the threshold in Experiment 2 (The interaction effect). (**a**) the simple main effect of materials within each viewing angle. (**b**) the simple main effect of materials within each light field. The left column in panels (**a**) and (**b**) shows the fitting curves based on the median of the threshold and slope of 10,000 repetitions by the bootstrapping method. The median threshold is shown in the top-left corner. The right columns in panels (a) and (b) show the distributions of the threshold in each condition estimated by the bootstrapping method. The black bar and white dot inside the violin plot indicate the interquartile range [Q1, Q3] and median [Q2], respectively. For threshold difference, *** *p* < 0.001; ** *p* < 0.01; * *p* < 0.05 after Bonferroni-Holm correction.

The interaction between material and viewing angle or deformation type is shown in Figure 11. For the simple main effect of materials within each viewing angle. In the 0° viewing condition, the transparent material (*Median* = 4.53) has a higher threshold than dotted matte (*Median* = 4.10, *p* = 0.0075), glossy (*Median* = 4.11, *p* = 0.0024) and mirror (*Median* = 4.22, *p* = 0.0212). In the 45° viewing condition, the transparent material has a higher threshold than dotted matte (*p* = 0.0018). For the simple main effect of materials within each deformation type, the original squeeze condition showed that the transparent has a higher threshold than dotted matte, glossy, and mirror (*p*s < 0.0001). In the Jiggle condition, the transparent has a higher threshold than dotted matte (*p* = 0.0162). There is no significant difference among materials in the twist condition.

#### Generalized linear mixed model for accuracy analysis

A GLMM logistic regression was conducted with the fixed effects of deformation intensity, viewing angle, deformation type, and material, and all of their higher-order interactions (Table 1). We also included a random intercept and slope per participant. Aside from the new predictor of deformation type, the effects observed were similar to those in the previous experiment. We found significant main effects of viewing angle (*χ*^2^ = 87.18, *p* < 0.0001) and deformation intensity (*χ*^2^ = 176.88, *p* < 0.0001). There was a significant main effect of material (*χ*^2^ = 14.43, *p* = 0.0024) and Tukey's HSD post hoc test showed that the transparent material was significantly different from the dotted matte (*z* = 4.09, *p* = 0.0003), glossy (*z* = 3.38, *p* = 0.0040) and mirror (z = 3.35, *p* = 0.0045). There was a significant main effect of deformation type (*χ*^2^ = 62.51, *p* < 0.0001), and Tukey's HSD post hoc test showed that the twist was significantly different from the squeeze (z = 8.68, *p* < 0.0001) and jiggle (*z* = 7.08, *p* < 0.0001). We also found a significant interaction between deformation type and viewing angle (*χ*^2^ = 16.55, *p* = 0.0003), whereas twist was significantly different from squeeze (0° viewing: *z* = 8.86, *p* < 0.0001; 45° viewing: *z* = 3.45, *p* = 0.0017) and jiggle (0° viewing: z = 6.51, *p* < 0.0001; 45° viewing: z = 4.79, *p* < 0.0001) in both viewing angles. On the other hand, jiggle versus squeeze showed similar results at 0° viewing (*z* = 1.26, *p* = 0.4168) and 45° viewing conditions (*z* = 1.85, *p* = 0.1528).

There was a significant interaction between deformation intensity and viewing angle (*χ*^2^ = 92.17, *p* < 0.0001), and Tukey's HSD post hoc test showed that the 45° viewing condition has a steeper slope as a function of deformation intensity than the 0° viewing condition (*z* = 9.75, *p* < 0.0001). There was a significant interaction between deformation intensity and material (*χ*^2^ = 13.88, *p* = 0.0031). The Tukey's HSD post hoc test showed that the slopes of the Transparent material were significantly shallower than the dotted matte (*z* = 3.29, *p* = 0.0056) and glossy (*z* = 3.23, *p* = 0.0067) materials. There was also a significant interaction between deformation intensity and deformation type (*χ*^2^ = 80.81, *p* < 0.0001). The Tukey's HSD post hoc test showed that the slopes of the twist deformation were significantly shallower than the squeeze (*z* = 5.99, *p* < 0.0001) and twist (*z* = 8.81, *p* < 0.0001). There were no other two-way or higher-order interactions.

### Discussion

The results indicated a diversity in the perception of deformation between the twist and squeeze/jiggle deformations. Furthermore, we also found that viewing angles and deformation types interact. This suggests that our overall performance is contingent upon the type of deformation observed, with a consistent reduction in ability when faced with a more challenging viewing angle, albeit varying in magnitude depending on the deformation type.

The most notable finding was the lack of a significant interaction between material and deformation type. Regardless of the deformation type, the deformation detection performance was affected little by material, or only slightly worse for Transparent material than for the other materials. This implies that the human visual system exhibits resilience against alterations in appearance from material changes in deformation detection.

## General discussion

The present results show that deformation detection performance is affected by material changes. Deformations are harder to detect with transparent materials than with matte or glossy materials. However, the difference in detection thresholds was not large. In addition, the effects of material are affected little by the viewing angle (which itself affected significantly the deformation threshold), light field (which itself had little effect on the deformation threshold), and deformation type. Note that the responses were collected without feedback, making it unlikely that participants learned to rely on trivial image cues that were valid only for a specific type of stimulus condition during the deformation detection task.

### Optical flow analysis

**Figure 12.**
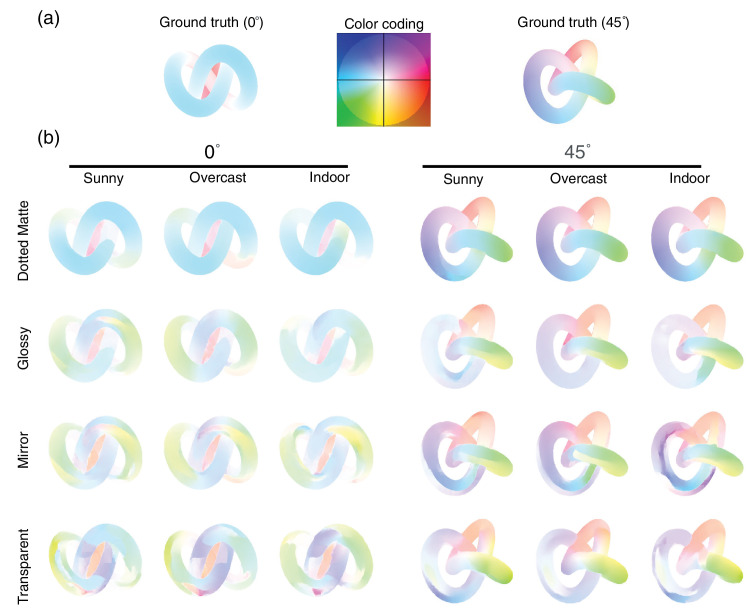
The optical flows of the squeeze distortion stimuli used in both experiments. Shown examples are those computed for the transition from the 1st frame to the 2nd frame. (**a**) The ground truth optical flow under 0° viewing (left) and 45° viewing (right) conditions. The central panel indicates the color coding for optical flow (central), where saturation indicates motion speed while hue motion direction. (**b**) The RAFT-estimated optical flows for different combinations of light fields (columns) and materials (rows) under 0° viewing (left) and 45° viewing (right) conditions.

To gain insights into the underlying computation for deformation detection, we analyzed how the spatiotemporal pattern of image motion (optical flow) is affected by the stimulus manipulation for the “squeeze” deformation stimuli used in both experiments. We did this by comparing the ground truth flow (true motion unaffected by optical effects) and estimated optical flow (apparent motion influenced by optical effects). (1) To calculate the ground truth flow, we took the Cartesian distance between the (x,y,z) vertices of the object files used to render consecutive frames. Using OpenGL, we determined visible and occluded vertices in the experimental stimuli by using the same settings as in the rendering and considering the motion of visible vertices only. (2) To estimate the image motion flow, we used a recent deep neural network for optical flow estimator, the Recurrent All-pairs Field Transforms (RAFT) model (Teed & Deng, 2020). Since the input given to RAFT is identical to that given to the human participants, we expected the RAFT's output to be an estimate of the image optical flow they saw. This, however, is only a rough estimate since RAFT cannot accurately predict human perceived flow under some conditions (Sun, Chen, Yang, & Nishida, 2023a; Sun, Chen, Yang, & Nishida, 2023b; Yang, Fukiage, Sun, & Nishida, 2023). Sequences of flows are obtained by processing all pairs of consecutive frames. The output for each consecutive pair was a 512 × 512 × 2 matrix that contains the vertical and horizontal motion for that pixel from the current frame to the next frame. We then transformed this into polar coordinates, with a direction and speed component, as shown in Figure 12, and dynamic examples of the stimuli are available in the supplementary materials (Supplementary Video S4). The similarity of the optical flow between different stimulus conditions, estimated by the correlation, is summarized in Table 2.

**Table 2a.** The cross-correlations of the optical flows of the squeeze deformation stimuli. For each cell, the shown value was the Pearson correlation using the horizontal and vertical components of the local motion vector at all spatial locations (except for the background) in all 120 frames. The correlations between materials (four materials and the GT) for two viewing angles and three light fields.

	GT	Dotted matte	Glossy	Mirror	Transparent
Sunny					
0°					
GT	1.000				
Dotted Matte	0.920	1.000			
Glossy	0.717	0.765	1.000		
Mirror	0.643	0.692	0.933	1.000	
Transparent	0.684	0.721	0.843	0.823	1.000
45°					
GT	1.000				
Dotted Matte	0.965	1.000			
Glossy	0.852	0.874	1.000		
Mirror	0.831	0.851	0.935	1.000	
Transparent	0.774	0.797	0.816	0.792	1.000
Overcast					
0°					
GT	1.000				
Dotted Matte	0.916	1.000			
Glossy	0.738	0.790	1.000		
Mirror	0.680	0.735	0.946	1.000	
Transparent	0.684	0.724	0.874	0.852	1.000
45°					
GT	1.000				
Dotted Matte	0.967	1.000			
Glossy	0.897	0.921	1.000		
Mirror	0.856	0.877	0.938	1.000	
Transparent	0.795	0.822	0.852	0.794	1.000
Indoor					
0°					
GT	1.000				
Dotted Matte	0.923	1.000			
Glossy	0.728	0.777	1.000		
Mirror	0.616	0.655	0.853	1.000	
Transparent	0.650	0.669	0.804	0.810	1.000
45°					
GT	1.000				
Dotted Matte	0.960	1.000			
Glossy	0.848	0.881	1.000		
Mirror	0.809	0.834	0.898	1.000	
Transparent	0.768	0.792	0.786	0.757	1.000

Under the 0° viewing condition, the ground truth flow predominantly consisted of horizontal directions produced by the rotation along the vertical axis. Under the 45° viewing condition, the flow includes a wide range of directions due to the rotation along slanted axes relative to the viewer.

The estimated optical flow is influenced very little by light field, as evidenced by high correlations between completely different light fields (see Table 2b). Little change in optical flow by light field is consistent with our finding that changing light field had little effect on deformation detection performance.

**Table 2b.** The cross-correlations of the optical flows of the squeeze deformation stimuli. For each cell, the shown value was the Pearson correlation using the horizontal and vertical components of the local motion vector at all spatial locations (except for the background) in all 120 frames. The correlations between light fields for two viewing directions and four materials.

	Sunny	Overcast	Indoor
Dotted Matte			
0°			
Sunny	1.000		
Overcast	0.970	1.000	
Indoor	0.960	0.960	1.000
45°			
Sunny	1.000		
Overcast	0.989	1.000	
Indoor	0.984	0.984	1.000
Glossy			
0°			
Sunny	1.000		
Overcast	0.935	1.000	
Indoor	0.899	0.895	1.000
45°			
Sunny	1.000		
Overcast	0.925	1.000	
Indoor	0.897	0.917	1.000
Mirror			
0°			
Sunny	1.000		
Overcast	0.930	1.000	
Indoor	0.900	0.900	1.000
45°			
Sunny	1.000		
Overcast	0.922	1.000	
Indoor	0.912	0.911	1.000
Transparent			
0°			
Sunny	1.000		
Overcast	0.922	1.000	
Indoor	0.896	0.909	1.000
45°			
Sunny	1.000		
Overcast	0.949	1.000	
Indoor	0.920	0.926	1.000

On the other hand, the estimated optical flow is changed by materials. Dotted matte material, the only material in our set without reflective or refractive components, is similar to the ground truth (*r* = 0.92, see Table 2a). The similarity to the ground truth flow, however, is reduced for glossy, and further reduced for mirror and transparent. The observation that transparent is one of the stimuli least similar to the ground truth seems consistent with our finding that deformation detection performance was worse for this material. However, this factor alone cannot explain why glossy and mirror materials are nearly as good in deformation detection as Dotted matte.

**Figure 13.**
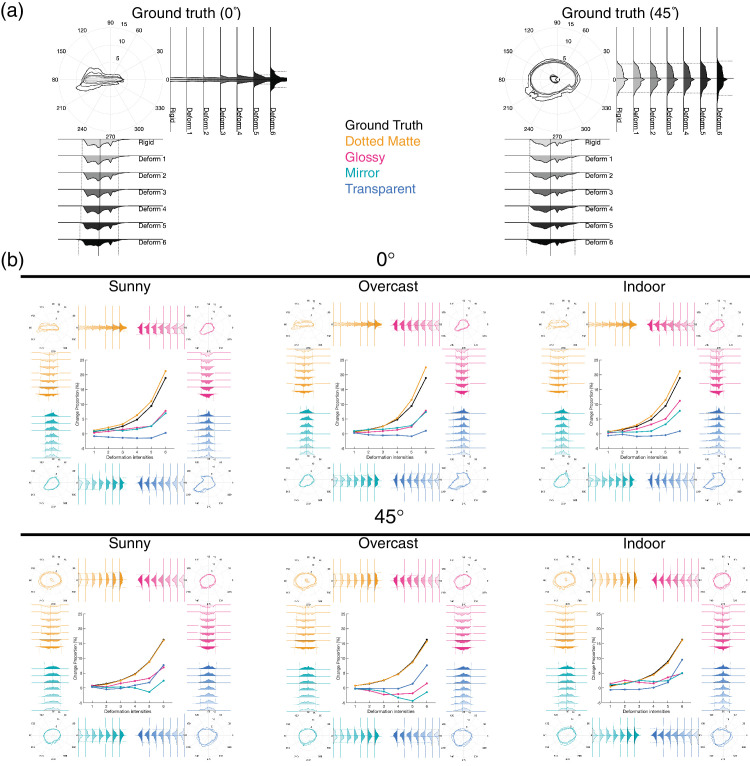
Analyses of the optical flow of the squeeze stimuli. (**a**) The ground truth optical flow at different deformation magnitudes under 0° viewing (left) and 45° viewing (right) conditions. In each panel, the contour plot indicates the range of 95% of the flow vector distribution for each deformation intensity, where the angle encodes the direction and the radius encodes the speed. The histograms represent the marginal distribution of the horizontal and vertical components of the optical flow with the mean (solid lines) and ±1.96SD (dashed lines), indicating the main trend of their distribution. (**b**) Each panel shows the RAFT estimated optical flows for different combinations of light field (columns) and viewing angle (rows). At each corner of each panel, a 2D contour plot and marginal distribution histograms are shown for each material and deformation magnitude. The center of each panel shows how the variation of optical flow, estimated as the mean standard deviation of the optical flows (normalized by that of the rigid stimuli), changes as a function of deformation magnitude in each condition. Note that the ground truth is identical across illuminations.

To better understand how image motion changes with deformation, material, light field, and viewing angle, we visualize the statistics of the ground truth and RAFT-estimated optical flow of the Squeeze deformation stimuli in Figure 13. The contour plots and histograms show the 2D distribution and the one-dimensional (1D) marginal distributions of the vertical and horizontal components of the local optical flow with 1.96 SD (dashed lines in 1D histograms) to represent the main trend of their distribution. The ground truth distribution (Figure 13a) indicates that the flow distribution is broadened as the deformation magnitude is increased, which is naturally expected from the way we deformed the image. Figure 13b shows how the image flow statistics are modulated by material, light field, and viewing angle. In addition to showing the flow distributions, we show the change in the standard deviation (SD) of the optical flows at the center of each panel in Figure 13b. To achieve this, we initially computed the SD of the optical flow by using the aforementioned 512 × 512 × 2 optical flow matrix across 120 frames for each experimental condition and deformation intensity. Then, to standardize the comparisons, we normalized these SD values by dividing them by the SD of the optical flow for the rigid stimuli in each respective condition. This normalization process ensured that we could effectively compare the variations in optical flow across different deformation intensities and conditions.

Regarding the ground truth flow, the geometrical Squeeze deformation of an object produces additional motion (in the vertical direction for the 0° viewing condition) compared to the flow produced by the rigid motion of the object. Therefore the flow SD is a candidate for a simple image statistic correlated with the deformation magnitude. Indeed, the flow SD of the ground truth increases with the increase in deformation magnitude regardless of optical conditions. Regarding the RAFT-estimated flow, the same trend is also found for the dotted matte material. However, the other materials showed a much slower increase, or non-monotonic change, in the SD with the deformation magnitude. This can be ascribed to the specular reflection and transparent refraction producing non-smooth complex flow even for rigid rotation of our knot object. In other words, the variation in optical flow produced by deformation is masked by those produced by complex motion of specular reflection and transparent refraction. These flow statistics may be able to explain why deformation detection is difficult for transparent materials. However, these statistics predicted a stronger material effect than what we observed in the perceived deformation. This finding implies that while transparent refraction significantly affects optical flow, it does not influence perceived deformation as much as the flow statistics would imply.

### Possible underlying computation

The above analysis suggests that deformation detection would be seriously affected by the surface material if human observers use simple image features in the flow statistics, such as an increase in the variance of motion flow, or some features correlated with it, regardless of what material the object is made of. Inconsistent with this, we found material-robust detection of deformation. What kind of visual computation does this finding indicate?

One possibility is that the human brain judges the object's (non-) rigidity based on image features and/or computations specific to each material. It is known that surface flow patterns could be a visual cue to judge material (Doerschner, Kersten, & Schrater, 2011; Doerschner, Fleming, et al., 2011; Tamura et al., 2018; Yilmaz & Doerschner, 2014). Once the material parameters are specified by the flow pattern or other image cues, then there should be a way to judge whether the flow is consistent with that of a rigid object or not. In this computation, the visual system may use a generative model that predicts how the motion flow should change over time if the object of a specific material is rigidly moving, and judges the object as non-rigid if the observed flow significantly deviated from the predicted rigid motion (Yildiran et al., 2023).

Another possibility is that the human brain judges the object's (non-)rigidity mainly on material-invariant features, while ignoring other image flow information as “noise.” The third possibility is the hybrid of the two hypotheses such that the human brain judges the object's (non-)rigidity based both on material-dependent and independent image features.

Further examination of these hypotheses is underway. Specifically, object boundary, or silhouette, is a typical feature affected little by material. The dynamic changes of an object's silhouette contains significant information about the 3D structure of the object (Cheung, Baker, & Kanade, 2005; Giblin, 1987; Pollick, Giblin, Rycroft, & Wilson, 1992) and humans can perceive rigid and non-rigid 3D structures solely from silhouette changes (Norman & Todd, 1994; Pollick et al., 1992; Wallach & O'Connell, 1953. Therefore our ongoing study examines how well human observers can detect deformation solely from dynamic silhouette information and how the performance is affected by the image flow within the object boundary.

### Limitations

Although we tested a range of materials and light fields, we used only a knot object. This complex shape possesses several basic shape properties, but the extent to which the current findings can be generalized to other shapes remains uncertain. Furthermore, we tested three types of deformation; however, they represent only a limited subset, where many other forms remain unexplored. In addition, our study does not explore multiple rotation axes or occlusion effects, which may influence shape perception (Dövencioǧlu et al., 2017). Although our findings suggest that global motion alone is not sufficient for deformation perception and that material properties play a role, further research is needed to disentangle the complex interactions between global and local motion. The range of possible deformation interactions is vast and intricate, making it challenging to untangle all the facets that may influence our judgments.

### Conclusions

Our results show that although deformation detection is slightly influenced by changes in material, it remains almost stable even amid significant variations in image motion. These results provide insights into the remarkable capacity of the human visual system to perceive dynamic 3D structures in natural scenes. This ability may be facilitated by neural mechanisms that understand how images should move for specular, mirror, and transparent materials, or mechanisms that effectively use material-invariant features indicating object deformations.

## Supplementary Material

Supplement 1

Supplement 2

Supplement 3

Supplement 4

Supplement 5

## Footnotes

1 A region in space in which the force is still present, but decreases in strength relative to the distance of the force-center.
