# Supplementary material for: Optical material properties affect detection of deformation of non-rigid rotating objects, but only slightly
Source: J Vis. 2025 May 12;25(6):6. doi: 10.1167/jov.25.6.6 (PMC12080736; doi:10.1167/jov.25.6.6)
Supplement: Supplement 1 [file jovi-25-6-6_s001.pdf]

Optical material properties affect detection of deformation of non-rigid rotating objects, but only slightly

Mitchell J. P. van Zuijlen[a], Yung-Hao Yang[a], Jan Jaap R. van Assen[b], Shin'ya Nishida[a,*]

[a]Cognitive Informatics Lab, Graduate School of Informatics, Kyoto University, Kyoto, Japan
[b]Perceptual Intelligence Lab, Faculty of Industrial Design Engineering, Delft University of Technology, Delft, The Netherlands

---

Appendix A. A Preliminary experiment

In a preliminary experiment, we used ten types of materials and two types of psychophysical tasks to select the proper methods for the main experiments. We also checked whether the range of deformation intensities was appropriate.

Appendix A.1. Methods

Appendix A.1.1. Participants

The experiment involved eight participants, consisting of six naive lab members and two of the study's authors, with a gender distribution of six males and two females. All participants had normal or corrected- to-normal vision and provided informed consent prior to participation in the experiment.

Appendix A.1.2. Stimuli and Design

In addition to the four optical materials (i.e., Dotted Matte, Glossy, Mirror, and Transparent) to be used in the main experiments, this experiment tested six optical materials: Anisotropic, Matte, Satin, Glossy and Matte texture, Translucent, and Transparent (reflective index: diamond). In total, we created a set of 10 materials, which are listed and visualized in Figure A1. We tried to create a set that contained the major material types, including matte, glossy, translucent, and transparent materials, drawing on our experience of working with materials. For the two transparent materials, the reflective index of the material matches that of water or diamond.

To reduce the number of trials, we did not use deformation intensity 0 (i.e., rigid, non deforming) and the deformation intensity 6 (i.e., the greatest deformation) as we expected that deformation intensity 1 would still be considered as rigid by participants and that deformation intensity 5 would likely already be very easy. Nevertheless, we included two trials at deformation intensity 6 (with a random material and random illumination) to serve as catch trials. This makes for a total number of stimuli equal to 152 stimuli = 10 materials * 3 light fields * 5 deformations + 2 catch stimuli. Each of 152 stimuli consisted of 120 frames, for a total of 18240 unique frames.

The order of the frames (normal or reversed) was randomly determined at the start of each trial. In the normal order, the stimuli were observed rotating clockwise, while they rotated counterclockwise in the reversed order. Additionally, the initial frame for each stimulus was also randomly selected at the beginning of each presentation. This means that the presentation of stimuli did not necessarily commence with the first frame but could initiate at any randomly chosen frame within the range of 1 to 120, and the subsequent frames

would follow in sequential order.

Appendix A.1.3. Procedure

This preliminary experiment used two tasks (Yes-no task and Two interval forced choice task, see below) within a single session, and the order of the two tasks was counter-balanced.

Yes-no task. In each trial, participants were presented with a single stimulus for each of the 152 trials (i.e., varying across 5 levels of deformation, 10 materials, and 3 illumination conditions, and two catch trials). All 120 frames for each stimulus were presented at 60 frames per second, for a total stimuli presentation time of two seconds. Participants were asked if the stimuli displayed deformation or not. Note that the stimuli did not include the non-deforming rigid condition, and thus perfect performance could be reached by simply responding "yes" in every trial. Stimuli were presented in four blocks of 38 stimuli, with a 2-second inter-trial time and a 45-second break between blocks.

Two interval forced choice (2-IFC) task. The other task was a 2-IFC task, where the participants were shown a reference and a target stimulus in each trial. Both stimuli were presented at 60 frames per second, for a stimulus presentation time of two seconds, and were separated by a 1 second inter stimuli interval (ISI). The target stimuli would be 152 stimuli described above, and the reference stimuli would always be presented at deformation intensity 1, i.e., the lowest level of deformation used within this preliminary experiment. The material and illumination condition for the reference stimuli would always be identical to that of the target stimuli. As such, the target and reference could only vary on deformation intensity. Note that the target stimuli would be presented at deformation intensity 1, and thus the target and reference stimuli would be identical.

Due to computer memory limitations (which were addressed before conducting the main experiments), we could not load all the 18240 images prior to the start of the experiment. As such, we split the 152 stimuli into 4 blocks of 38 stimuli and loaded the required images per block into memory during a 45-second break in between blocks.

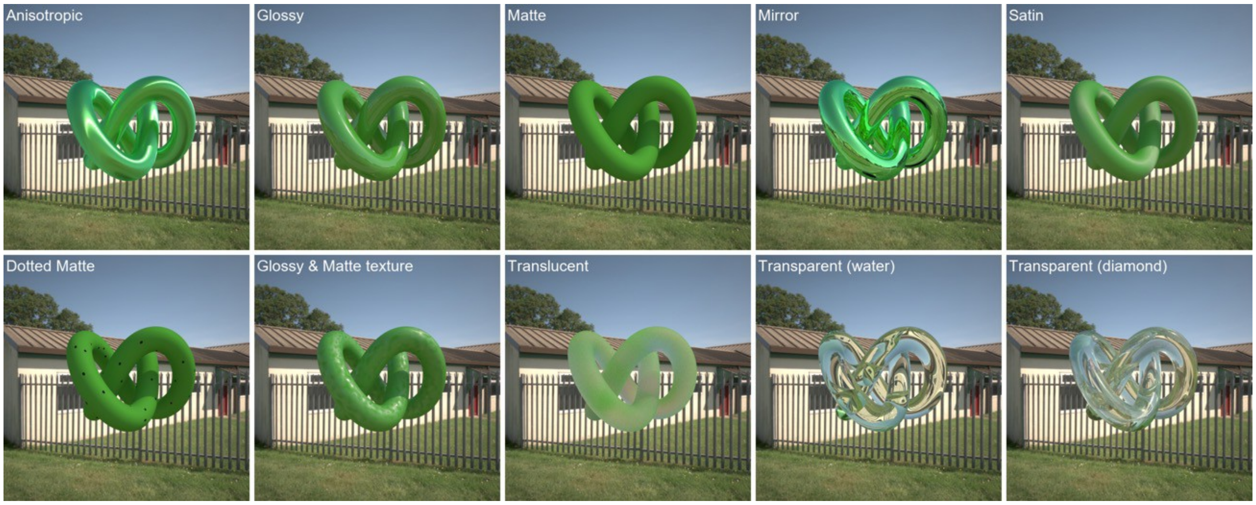

Figure A1: The 10 optical materials used in the preliminary experiment.

Appendix A.2. Results and Discussion

First, we looked at overall performance averaged across materials and illumination conditions. We did not apply any statistical testing on this data as we only collected one repetition per trial, and thus providing very little statistical power. Note that the two catch trials at deformation intensity 6 are not visualized here, as they were all correctly performed by all participants and shall not be included in any further analysis. Across both tasks, we can see that increasing deformation intensities improves performance. Figure A2(a) shows the responses for the yes-no task, we can see that a large deformation intensity is required before participants report perceiving a deformation. The findings indicate that when there is minimal deformation, participants only report perceiving deformation in roughly 10-15% of the trials. This suggests that participants tend to perceive objects as non-deforming, unless a clear deformation is perceived. Consequently, in the yes-no task with low deformation levels, neither of the stimuli is typically perceived as undergoing deformation.

Figure A2 (d) showed the performance of the 2IFC task, it should be noted that in 20% of the trials both the target and reference stimuli were at deformation intensity 1, and there was thus no correct answer. Instead, in the analysis, we randomly selected one of the two stimuli as the ’correct’ choice. As such, for these trials, the accuracy should approximate 50% and any deviation can be considered bias. We can see that performance is only just above the chance level for the lowest deformation intensity, while nearing a ceiling effect at the highest deformation intensity. This implies that the current range of deformation intensities is suitable to measure the perception of deformation, at least for the current set of stimuli and presentation conditions.

When averaging performance by material, as in Figure A2 (b) and Figure A2 (e), we can observe some difference in performance across materials, especially we can note that the materials with the transparent (diamond) component led to the lowest performance. Note, however, the large size of the 95% confidence interval error bars; based on this data we should not make any interpretations on significant differences across materials, nor across illuminations Figure A2 (c) and Figure A2 (f). From this preliminary experiment we conclude that 1) participants are able to perform the task, 2) the perceptual range of deformations appears appropriate, i.e., performance at/near chance for the lowest deformation and near perfect performance at the highest deformation. Taken together, we have validated our task and range of deformations for the main experiments. While the absolute magnitude of performance differs across the two tasks, the patterns appear quite similar. As such, we decided to continue with the 2IFC task for the main experiment, as we expect that experimental data on deformation discrimination (2IFC) is more informative than deformation detection (yes/no).

For the optical materials used in the experiment, we selected a set as small as possible (to reduce trials and the total experiment duration), while simultaneously as informative as possible. As such, this selection was somewhat arbitrary by nature and requires the use of assumptions. We settled on a set of four optical materials. These were Dotted Matte, Glossy, Mirror and Transparent (Water) referred to as Transparent in later sections. This refraction component would also be included for the Transparent (Diamond) material, which makes the choice between these two transparent materials again somewhat arbitrary. We opted for the transparent materials whose reflective indexed matches water, assuming participants might be slightly more familiar with this material.

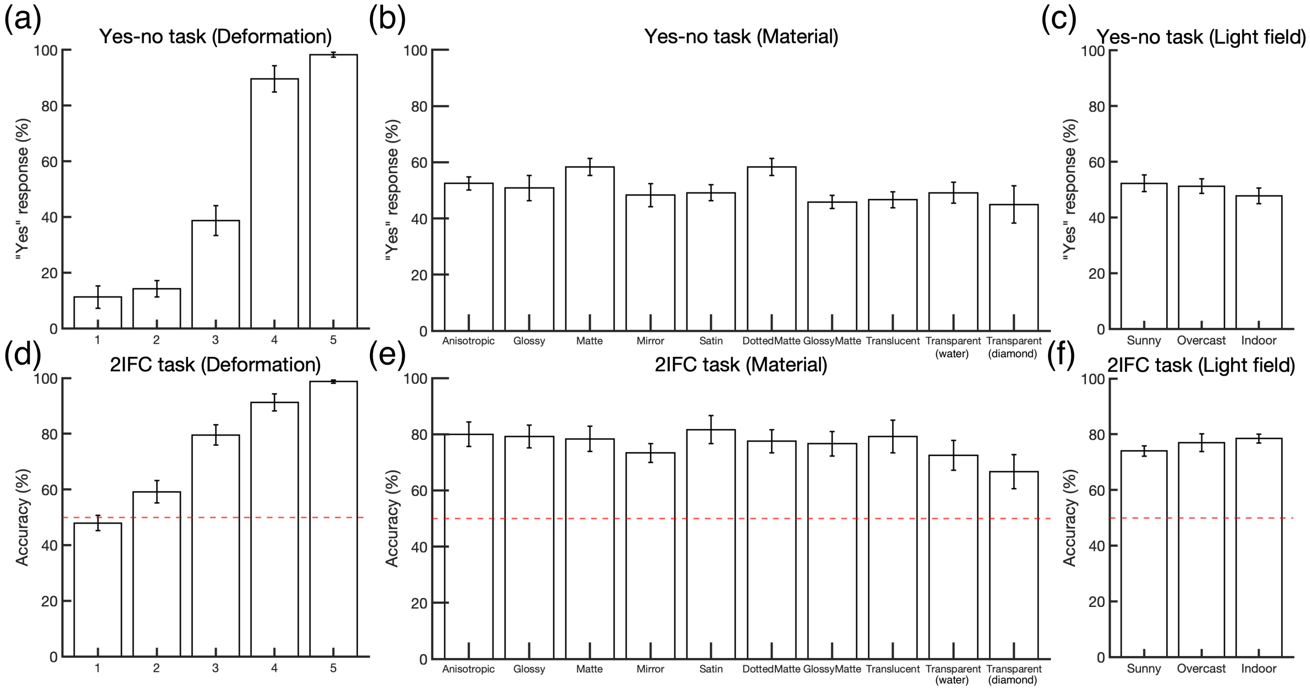

Figure A2: The results of the preliminary experiment. The top panels reveal the performance of the yes-no task, and the bottom panels reveal the performance of the 2-IFC task. Panels from left to right show the performance as a function of deformation intensities, materials, and illuminations, respectively.
